# Illustrations of the Influence of the Mind upon the Body in Health and Disease, Designed to Elucidate the Action of the Imagination

**Published:** 1884-11

**Authors:** 


ILLUSTRATIONS OF THE INFLUENCE OF THE MIND UPON THE BODY IN HEALTH AND DISEASE, DESIGNED TO ELUCIDATE THE ACTION OF THE IMAGINATION. *By* DANIEL HACK TUKE, M.D., L.L.D., ETC. *Second American, from the second English Edition. 8 vo., cloth, pp. 482. Philadelphia:* HENRY C. LEA'S SON & CO. 1884. *Chicago:* S. A. MAXWELL & CO.

In this portly volume, Dr. Tuke, under the modest intention of giving a collection of authenticated facts, in which the imagination has worked wonders, for good and evil, in the human economy, enters into the mechanical laws by which such facts are produced, and gives what, to unbiased men, will appear a thorough explanation of the miracles performed of old by the Mystics and by the Spiritualists in our days. Nothing has been omitted, not even the rationale of *stigmatization.*

The large amount of interesting anecdotes may render this book popular with the profession, the educated classes, and even the commonest readers. This could not be the case if the philosophy of the subject had been more deeply inquired into, but such a treatment of the subject would not have given the author as positive views as he derives from pure physiology, and to the majority of readers his explanations will appear, for that very reason, the most satisfactory.

The Psycho-Therapeutics, which are the outcome of the facts stated by the author, are worthy of careful study. In the use of doubtful agents, as electricity, for instance, it is sometimes imperative to give the patient the strongest hopes. Fear is not recommended as a safe agent. The strengthening of the will in hysteria will be beneficial. Systematic excitement of a definite expectation, direction of the attention to a particular region of the body, mesmerism, braidism, etc., have their range of utility, with some extrinsic value, and should be more generally known by physicians. This volume is neatly printed. It contains a few explanatory diagrams, and is highly interesting throughout. It is destined to eliminate many religious superstitions among the learned classes, and will contribute some sound information to the busy practitioner, but the quacks will, financially, make the most of it. H. D. V.

